# The Analysis of Cumulative Influence of Factors of Environment on a State of Health of the Population of Vladimir Region

**DOI:** 10.5539/gjhs.v7n3p309

**Published:** 2015-01-14

**Authors:** Tatyana Anatolyevna Trifonova, Leonid Alekseevich Shirkin

**Affiliations:** 1 Vladimir State University, Russia, 600000, Vladimir, Gorky Street, 87

**Keywords:** blood circulatory system, incidence, factors of environment, health state, adult population

## Abstract

There was investigated the contribution of factors of environment to formation of health for adult population on indicators of mid-annual rates of growth/decrease of disease of system of blood circulation and of some interfaced nosology on an example of the population of Vladimir region. The differential criterion of primary disease of system of blood circulation is considered as an indicator, integrally reflecting degree of adaptation to environment conditions on population and suitable for construction short-term prognostic estimations.

It is shown that business factors or the factors of a standard of living characterized by economic indicators, are leading risk factors in disease of system of blood circulation in Vladimir region which contribution is estimated by size of 38 %.

With use of regressive equations were received look-ahead estimations of annual rates of primary disease of system of blood circulation. In the regional centre Vladimir was observed more intense situation on rates of disease of system of blood circulation, than in Vladimir region.

## 1. Introduction

The circulatory system diseases are the number one cause of deaths worldwide. For the past decade, the mortality from cardiovascular diseases in the Russian Federation and the countries of Eastern Europe has significantly exceeded the same indicator in the Western countries, and led to reduction in life expectancy of the population of the Russian Federation (Rayner, Allender, Scarborough et al., 2009). Analysis of causes of deaths from the blood circulatory system diseases showed that the ischemic heart disease is on the first place (48.1%), followed by the cerebrovascular diseases (36.7%), their share equals to 84.8% of all deaths in this class.

It is generally accepted that human health is determined by the complex impact of a number of factors: heredity, way and quality of life, as well as the quality of the environment. The aggregate impact on the population’s health consists of the lifestyle (50%), the habitat (20%), the heredity (20%), and the quality of healthcare (10%). But these data are indicative and may vary significantly in different regions (Levi et al., 2009). The increase in negative manifestations in the economic and socioeconomic spheres leads to changes in the levels of adaptation of the population as a whole: from pre-disease states to formation of pathology (O’Keefe, 2009).

It is obvious that there often is complex effect of factors leading to occurrence of pathologies, especially typical of major towns and cities. From this perspective, cardiovascular diseases require special focus (WHO, 2013; Archana Singh-Manoux et al., 2010). Over the past 40 years, the proportion of cardiovascular diseases in the structure of mortality in Russia at various periods exceeded 50% of all deaths (WHO, 2013; O’Keefe et al., 2009; Panico, 2010).

The essential prognostic importance for methods of regional medical-ecological diagnostics is the condition of cardiovascular system, considered by researchers as the most informative indicator of adaptable possibilities of an organism possesses (Tulchinsky & Varavikova, 1999). Although, it is known that the age limit of mortality from these abnormalities can vary in various countries. The cardiovascular system, in addition to fulfillment of the hydrodynamic functions, plays the role of a matching link between the mechanisms of regulation and information and the morphological structures of the organism. On the one hand, these diseases are often associated with genetically determined abnormalities of the circulatory system, but on the other hand, they can become a consequence of other diseases caused by the negative pressure of the factors of the habitat or lifestyle of an individual or entire population. Therefore, the state of the cardiovascular system can be considered as the most informative indicator of the adaptive capacity of an organism.

There are currently no universally accepted data on the share of different factors in the formation of individual and population health, as the average share of the influence of individual factors on the health status may vary in different territories. And since the people’s living conditions are different in different regions, it is clear that the analytical and forward-looking estimates are useful if carried out for specific areas (Trifonova, 2009).

The purpose of the present research was revealing of the contribution of factors of environment in formation of adult population health on indicators of disease of system of blood circulation on an example of Vladimir region.

## 2. Objects and Methods of the Research

### 2.1 Objects

The object of the study was the population of the Vladimir region. The Vladimir region is located in the center of the European part of Russia, bounded on the west and south-west with the Moscow region, in the north – with the Yaroslavl and Ivanovo regions, in the south – with the Ryazan region, and in the east – with the Nizhny Novgorod region. The population of the region is 1.4 million; the population density is 48.59 people per km^2^. The share of the urban population is estimated at 78.1%. The central place in the regional economy is held by the industry. In the structure of the industry, engineering and metalworking prevail creating up to 40% of the industrial product; the food industry is rather significant (up to 17% of the industrial product), as well as the electric energy industry (10%), the glass production (up to 7%) and the light industry (about 5%).

The factors of environment were investigated which were directly connected with illnesses of system of blood circulation, in Vladimir’s territory and Vladimir region as a whole.

For the analysis, we used the statistical materials for 2003-2008: 1) on the primary disease incidence of the adult population, collected and published in the collection “The health status of the population of the Vladimir region” by the State Budget Institution of Healthcare of Special Type of the Vladimir region “Medical Information and Analytical Center” (SBIH VR “MIAC”, 2003-2008), 2) on the living standards of the population from official statistical publications of the Federal State Statistics Service Office in the Vladimir region (Vladimirstat, 2003-2008). The selection covered all 19 districts of the Vladimir region. Normally, the generated statistical selections in the medical and environmental studies should cover a 5-10-year period [Archana Singh-Manoux et al., 2010]. This study used statistical selection for the Vladimir region, formed for the 6-year period of observation.

### 2.2 Research Methods

As a basis for risk assessment, we adopted the classification of factors used by WHO with account of individual parameters of the population’s lifestyle, environment, heredity, and quality of healthcare. Various diseases have obvious links to the specified features of life of the population and are supported by these risk factors. It is known that lifestyle includes economic, sociological, socio-psychological, and socioeconomic factors, the quantitative estimation of the impact of which at the population level does not always have unambiguous simple solutions. We have chosen the most significant, in our view, positions for each of the factors, which may be characterized by indirect quantitative criteria.

For example, the role of economic factors, conditions, and lifestyle of the population is highlighted by the great differences in morbidity and mortality of the coronary heart disease among various social, material, and often national layers and groups of the population (Diez-Roux A.V., Northridge M.E., Morabia A., et al, 1999; Ruiz-Ramos M., 2008). People in countries with low and medium income are more exposed to risk factors, such as tobacco, which result in development of CVD and other non-communicable diseases (WHO, 2013). Therefore, the present study treats the *growth rate of the financial wealth of the population (the nominal gross wages and salaries)* as the leading factor among *the economic factors*.

Psychosocial factors are the marital status, stress, low social support, education, profession, depression, anxiety, hostility, and somatic disorders. Psychosocial factors are attached great importance, though it remains unknown how these factors influence on the growth of mortality from cardiovascular diseases – either directly or through other risk factors (Panico, S., 2010). Psychosocial stress turns out to be a factor of the risk of cardiovascular diseases, irrespective of ethnic and geographical context, so we consider *the average annual rate of incidence of the nervous system diseases* (G00 - G99) to be the indirect quantitative criterion of the effect of cumulative impact of *social* and *socio-psychological* *factors of risk*.

The *average annual rate of injuries and intoxication* (S00 – T98) are considered as a quantitative criterion of the effect of *socioeconomic* factors. A comparative analysis of the causes of deaths in the working-age showed that the mortality rate for both men and women is mainly determined by external causes (unnatural causes of death). In most countries, decrease in mortality from cardiovascular diseases was preceded and accompanied by favorable changes in the lifestyle and, in particular, reduction of the negative impact of socioeconomic factors (Tulchinsky, 1999).

*The incidence of respiratory diseases* (J00 - J99) is considered as direct quantitative indication of the efficiency *of environmental or* *habitat factors* (Tulchinsky, 1999). Some studies showed the influence of the “man-made” chemical factors on the basic parameters of the cardiac function. There are reasons to assume that the “man-made” chemicals (especially members of the phenol production cycle) can have a negative impact on the mechanisms of neural regulation of the cardiac function and metabolism in the myocardium, thus contributing to the accelerated development of the clinical forms of the cardiovascular pathology.

The *endocrine system functioning* is primarily linked to the genetic status of the organism, the state of *the immune system and the peculiar features of nutrition* of the population (Salim Yusuf, 2010). The consequences of unhealthy nutrition and lack of physical activity may result for some people in high blood pressure, elevated blood glucose, elevated blood lipids, as well as overweight and obesity (WHO, 2013).

The main risk factors for the diseases of heart and stroke are unhealthy nutrition, physical inactivity, and tobacco use. This behavior leads to 80% of the ischemic heart disease and cerebrovascular disease (WHO, 2013).

In order to identify the relationships between the risk factors and the incidence of the circulatory system diseases, we used the regression analysis of the data of *differential indexes* of the primary disease incidence, the environmental factors, and lifestyle of the population in different parts of the Vladimir region. The advantage of differential criteria, for example, the average annual rate of growth/decline of morbidity, against the values of morbidity or specific economic and socio-environmental indexes in the medical and environmental analysis is that, firstly, the differential criteria characterize the strength of the trend or the strength of the existing risk factors; secondly, they are more “protected” from random errors and outlying cases in the collected statistics than the indicators of primary of general disease incidence; thirdly, they help minimize the impact of time lags in the analysis of the incidence of non-communicable diseases of the population.

The average annual rate of growth/decline of the attribute in the dimensionless form is proposed to estimate by a perennial series of observations for the studied area according to a formula built on a ratio of the first derivative, calculated by the method of the least squares for the best approximation of the dynamics of the test indicator, and the median value of the same indicator for the observation period:


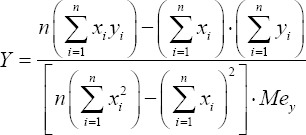


where *Y* is the average annual growth/decline of the index *y*, year^-1;^ *y_i_* is the value of the index for a particular year of measurement; *x_i_* is the year (or sequence number of the year in the selection), for which the value of the index is valid; *n* is the number of data in the row of observations; *Me_y_* is the median for the index *y* for the observation period – the value, which is in the middle of the series of ranked values.

To obtain aggregated prognostic estimates in terms of average annual rates of incidence of the circulatory system diseases, *the linear additive function* was used, the coefficients of which were obtained by *the regression analysis*. We considered as important factors: 1) the environmental factors, 2) the sociological and socio-psychological factors, 3) the factors of the nutritional status, 4) the economic factors, 5) the socioeconomic factors, 6) other factors, including the heredity factor.

The regression analysis of average annual rates of primary incidence of diseases of the circulatory system *z(f_1,_ f_2,_ f_3,_ f_4,_ f_5)_* was carried out according to the hyperplane equation:


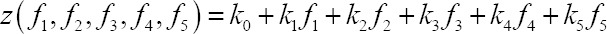


where *f_1_* is the annual rate of income growth (the economic factors), year^–1;^

*f_2_* is the annual rate of incidence of the respiratory system diseases (the environmental factors), year^–1;^

*f_3_* is the annual rate of injuries and intoxication (the socioeconomic factors), year^–1;^

*f_4_* is the annual rate of incidence of endocrine and nutritional diseases (the nutrition status factors), year^–1;^

*f_5_* is the annual rate of incidence of the nervous system diseases (the social and socio-psychological factors), year^–1;^

*k_0_* is the empirical coefficient that takes into account the cumulative effect of the factors permanently existing in the population, among which the leading ones are the hereditary factors in the incidence of cardiovascular diseases, year^–1;^

*k_1,_ k_2_*, *k_3,_* *k_4,_* *k_5_* are the empirical coefficients reflecting the effect of the risk factors existing in the population.

To find the coefficients *k_0,_* *k_1,_* *k_2,_* *k_3,_* *k_4,_* *k_5_*, the multivariate regression analysis was applied to the hyperplane function using the method of least squares and the Levenberg-Marquardt algorithm for optimization in the Mathcad environment. The accuracy of solutions was evaluated by the coefficient of determination.

## 3. Results

In the structure of the total mortality, the blood circulatory diseases in the Vladimir region accounted for 54.4% (2008), and these diseases for many years have been the number one cause of disability. The overall incidence of diseases of the circulatory system is also at a high level – 37,755.5 per 100 thousand people (in Russia – 26,415). Late appeal, late hospitalization do not allow for the necessary treatment, and, as a result, there is high mortality of up to 36% (WHO, 2013; O’Keefe J.H. et al., 2009; Panico S., 2010). Out of total morbidity of population in the Vladimir region, the diseases of the circulatory system hold the lead, followed by respiratory diseases (14%) and diseases of the musculoskeletal system, which are far behind.

Based on the collected statistical information for the 6-year period, we evaluated the differential criteria for indicators of primary incidence of diseases of the circulatory system. An example of calculation of differential indexes of primary disease incidence is provided for the population of the city of Vladimir (350 thousand people) and the population of the Vladimir region (1,413 thousand people) as a whole (Table 1).

**Table 1:** Estimate of average annual rate of growth/decline in terms of the primary incidence of diseases of the circulatory system (I00 – I99)

Population of the territories	Primary disease incidence per 1,000 of the adult population							Gradient of the trend line, year^–1^	Annual average rates, % a year

	2003	2004	2005	2006	2007	2008	Median line		
the city of Vladimir	16.6	18.6	18.3	20.4	17.6	19.9	18.5	0.4457	2.4
the Vladimir region	23.8	22.0	20.1	26.0	23.8	27.0	23.8	0.7800	3.3

Despite the fact that there has been observed the decrease in the primary disease incidence in some years, the calculated average annual rates of incidence of circulatory system diseases reflect the statistically significant upward trend for the adult population of the city of Vladimir, and the whole Vladimir region. The trend is characterized by a gradient of the trend line, or slope, which represents the amount, by which the average primary incidence of diseases of the circulatory system increases for one calendar year. The ratio of the gradient to the median value of the primary disease incidence allows estimating the average annual rate of growth/decline in percentage in terms of the primary incidence of the circulatory system diseases of the adult population (> 18 years).

Similarly, the differential criteria for indicators, which reflect the effect of the factors of nutritional status, the economic, environmental, socioeconomic, sociological and socio-psychological factors of risk, were calculated (Table 2).

**Table 2:** Estimate of the average annual rate of growth/decline of risk factors, %

Population of the territories	Annual income growth rate *(economic factors)*	Annual rate of the primary incidence of respiratory diseases *(the environmental factors)*	Annual rate of injuries and intoxication (*the socioeconomic factors*)	Annual rate of primary incidence of the endocrine and nutritional diseases *(the factors of nutritional status)*	Annual rate of primary incidence of the nervous system diseases *(the sociological and socio-psychological factors)*
the city of Vladimir	24.7	0.9	-1.5	21.3	5.7
the Vladimir region	23.4	-0.8	-1.4	9.1	-4.4

The multivariate regression analysis resulted in the following values of the empirical coefficients: *k_0_* = 0.186, *k_1_* = -0.555, *k_2_* = 0.327, *k_3_* = 0.622, *k_4_* = 0.028, *k_5_* = 0.332.

Thus, we obtained the following regression equation for the annual rate of growth/decline of the primary incidence of circulatory system diseases (z):


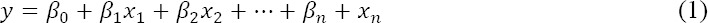


The coefficient of determination for the equation is *R* = 0.942. It shows what proportion of the variance of the resultant attribute is explained by the influence of independent variables *f_1,_ f_2_, f_3,_ f_4,_* and *f*_5_. The data in Table 2 served as the basis for receiving prognostic estimates.

## 4. Discussion

The performed study revealed the following regularities. The risk factors considered in the model show divergent trends in Vladimir and the Vladimir region (Table 2). In the Vladimir region, the incidence of endocrine and nutritional diseases tends to worsen, and the number of cases of injuries and intoxication tends to decrease. Significant differences between the city of Vladimir and the Vladimir region are observed in the annual rate of the primary incidence of respiratory diseases (the environmental factors), the annual rate of the primary incidence of the endocrine diseases (the factors of nutritional status), the annual rate of the primary incidence of the nervous system diseases (the sociological and socio-psychological factors).

By the sensitivity of the population’s health, i.e. by the ability of cardiovascular diseases incidence to respond to small impacts, the risk factors can be ranked in the order of decreasing empirical coefficients *k_i_* of the model as follows: 1) the socioeconomic factors – the lifestyle factors, which refer to the way of social life, everyday activity, culture, within which people carry out life-sustaining activity; 2) the economic factors – the factors of the living standards characterized by economic indicators (the amount and form of income, the consumption patterns, the housing quality and availability, and so on); 3) the sociological and socio-psychological factors – the factors of the quality of life, which are the assessment of the qualitative aspects of the living conditions (comfort) and the lifestyle factors that relate to the individual characteristics of behavior being one of manifestations of life activities; 4) the environmental factors; 5) the factors of nutritional status, estimated by annual rates of incidence of the endocrine and nutritional diseases.

By the relative contribution to the rate of incidence of the circulatory system diseases of the adult population of the Vladimir region, the risk factors can be ranked in the order of decreasing the index *k_i_ f_i_*/*z(f_1,_ f_2,_ f_3,_ f_4,_ f_5)_* as follows: 1) the economic factors characterized by the growth of income of the population; 2) the sociological and socio-psychological factors that are estimated by the rate of incidence of nervous system diseases; 3) the socioeconomic factors that are estimated by the annual rate of injuries and intoxication; 4) the environmental factors; 5) the factors of nutritional status. The economic factors are believed not to influence on the health directly, but they influence on it through the human behavior, i.e. indirectly (Ruiz-Ramos M., 2008). Nevertheless, more than 80% of deaths from cardiovascular diseases in the world occur in countries with low and medium level of income (WHO, 2011).

Predictive estimates of annual rates of primary incidence of diseases of the circulatory system were obtained for the city of Vladimir and the whole Vladimir region using the regression equation *z*(*f_1_, f_2_, f_3_, f_4_, f_5_*). The expected annual gain in the primary incidence of the circulatory system diseases for the adult population of the city of Vladimir is equal to 6.7%. The expected gain in the primary incidence of the circulatory system diseases for the next two years is equal to 13.8% if trends of the risk factors continue. The expected annual gain in the primary incidence of the circulatory system diseases for the adult population of the Vladimir region is equal to 3.3%. In this case, the expected gain in the primary incidence of the circulatory system diseases for the adult population for the next 2 years is 6.7 % if trends of the risk factors continue. In fact, during the subsequent 2009-2010, the Vladimir region demonstrated increase in the primary incidence of the circulatory system diseases at 8.6%.

The situation with the incidence of the circulatory system diseases in the regional center Vladimir is tenser than in the Vladimir region. The resulting estimates are statistically significant, because they exceed the minimum error value of 2% usually used in medical and statistical studies (Perricone R.A., 2009).

The use of the regression analysis for finding the primary function of the average annual rate of incidence of the circulatory system diseases *z(f_1,_ f_2,_ f_3,_ f_4,_ f_5_*) and the subsequent consolidated forecast estimates can be justified by the following provisions.

1). All information about the causes of the medical and environmental phenomenon development are contained in its implementation – in the levels and rates of the primary incidence of disease of the population for different nosological groups (Zueva, L.P., et al., 2009).
2). Identification of the relationship between the indicators of risk factors and the indicators of primary incidence of diseases of the adult population is legitimate, as the basis of such relationship is the non-specific influence on the organism of multiple causal factors of low intensity (Guidelines approved by the Chief State Sanitary Doctor of the Russian Federation on 30.07.1997 #2510/5716-97-32).
3). The cause and effect relationship between the factors, determining the health, and the health itself is the statistical relationship. The stronger the relationship between the presumed cause (the risk factor) and its effect is, the more likely the importance of this cause is (Guidelines approved by the Chief State Sanitary Doctor of the Russian Federation on 30.07.1997 #2510/5716-97-32).
4). Building a model of health and regression dependences based on the indicators reflecting the risk of reducing the functional state (adaptation) of the circulatory system is legitimate, because the criteria for health changes (reactions) of the population are discrete, i.e. there are people in the population who have different levels of adaptation – from pre-disease states (stress and overstress of adaptation) to the formation of pathology (Guidelines approved by the State Committee for Sanitary and Epidemiological Oversight of the Russian Federation on 26.02.1996 #01-19/12-17).

## 5. Conclusion

1). For analysis of the combined effects of medical and environmental factors on the health status of the region’s population, a methodology is proposed, which is based on identifying the relationship between the differential indexes of primary incidence of diseases of the blood circulatory system, the factors of the environment and lifestyle followed by building a model for the annual rate of the primary incidence of the circulatory system diseases. The differential criterion for primary incidence of the circulatory system diseases can be considered as the indicator integrally reflecting the degree of adaptation to the environmental conditions at the population level and suitable for building short-term prognostic estimates.
2). The peculiar features of the health status of an industrial center and non-urban population of the Vladimir region is determined by the significant differences in the effect of environmental factors, the factors of nutritional status, and the sociological and socio-psychological factors. The situation with the incidence of the circulatory system diseases in the regional center Vladimir is tenser than in the Vladimir region.
3). The expected annual gain in the primary incidence of the circulatory system diseases has been determined, which for the adult population of the Vladimir region is equal to 3.3 %.
4). The economic factors or the factors of the standard of living, characterized by economic indicators are the leading risk factors for the incidence of cardiovascular diseases in the Vladimir region, the contribution of which is estimated at 38%.

The study of the time series by the average annual rate of growth/decline of the indicator allows identifying trends in the incidence of the population of territories and characterizing the influence of the existing risk factors; conducting clustering of nondimensionalized indicators; and minimizing the impact of time lags and the impact of random fluctuations in the statistics.

The proposed approach to the analysis of the influence of environmental factors on the formation of population health for the adult population in terms of the incidence of the circulatory system diseases can be used to solve a number of problems, including:

–building the models of health and mathematical dependences that reflect the risk of deterioration of the functional state of organs and systems at the population level;
–evaluation of the contribution and ranking of risk factors;
–evaluation of the natural population-related and economic damage to the health caused by the premature loss of health in the active working age;
–development and comparative analysis of scenarios of reducing the natural population damage.

The proposed model is not able to study the nonlinear effects and the bifurcation mechanisms – it is intended for short-term forecasting (not exceeding 2 years) of medical and environmental processes, the dynamics of which are formed by the slow accumulation of new quantitative features.

## References

[ref1] Comprehensive hygienic assessment of the intensity of medical and environmental situation of various areas due to contamination by toxic environment of the population Guidelines approved by the Chief State Sanitary Doctor of the Russian Federation 30.07.1997 #2510/5716-97-32.

[ref2] Diez-Roux A.V, Northridge M.E, Morabia A (1999). Prevalence and social correlates of cardiovascular disease risk factors in Harlem. American journal of public health.

[ref3] Global status report on non-communicable diseases 2010 (2011). http://www.who.int/nmh/publications/ncd_report2010/en/.

[ref4] Guidelines “Uniform methods of data collection, analysis, and evaluation of morbidity with the complex action of environmental factors Approved by the State Committee for Sanitary and Epidemiological Oversight of the Russian Federation on 26.02.1996 #01-19/12-17. http://base.consultant.ru/cons/cgi/online.cgi?req=doc;base=EXP;n=366688.

[ref5] Levi F (2009). Mortality from cardiovascular and cerebrovascular diseases in Europe and other areas of the world: an update. European Journal of Cardiovascular Prevention and Rehabilitation.

[ref6] O'Keefe J. H, Carter M. D, Lavie C. J (2009). Primary and Secondary Prevention of Cardiovascular Diseases: A Practical Evidence-Based Approach. Mayo Clinic Proceedings.

[ref7] Panico S, Mattiello A (2010). Epidemiology of cardiovascular diseases in women in Europe. Nutrition, Metabolism and Cardiovascular Diseases.

[ref8] Perricone R. A (2009). The cerebro-vascular diseases' incidence in the cardio-circulatory out-line morbility's to implement prevention. Nutrition, Metabolism and Cardiovascular Diseases.

[ref9] Rayner M, Allender S, Scarborough P (2009). Cardiovascular disease in Europe. European Journal of Cardiovascular Prevention & Rehabilitation.

[ref10] Ruiz-Ramos M, Bono T. H, Anti-olo F. G (2008). Trends in mortality due to cardiovascular diseases in Andalusia, Spain (1975-2004). Revista Espanola de Salud Publica.

[ref11] Singh-Manoux A (2010). Lost work days in the 6 years leading to premature death from cardiovascular disease in men and women. Atherosclerosis, 211.

[ref12] The Federal State Statistics Service Office in the Vladimir region (n.d.). http://vladimirstat.gks.ru/wps/wcm/connect/rosstat_ts/vladimirstat/ru/publications/official_publications/electronic_versions/.

[ref13] Trifonova T. A, Salyakin I. E, Krasnoschekov A. N (2009). Evaluation of comfort of living of the population in the region using modern GIS technology. Ecological Systems and Devices.

[ref14] Tulchinsky T. H, Varavikova E. A (1999). The new public health: an introduction to modern science.

[ref15] World Health Organization (n.d). Cardiovascular diseases (CVDs). Fact sheet N°317.

[ref16] Yusuf S, Sonia A (2010). Deciphering the Causes of Cardiovascular and Other Complex Diseases in Populations: Achievements, Challenges, Opportunities, and Approaches. Progress in Cardiovascular Diseases.

[ref17] Zueva L. P, Eremin S. R, Aslan B. I (2009). Epidemiological diagnosis.

